# Post-epidemic Schmallenberg virus circulation: parallel bovine serological and *Culicoides* virological surveillance studies in Ireland

**DOI:** 10.1186/s12917-016-0865-7

**Published:** 2016-10-18

**Authors:** Á. B. Collins, D. Barrett, M. L. Doherty, M. Larska, J. F. Mee

**Affiliations:** 1 Animal and Bioscience Research Department, AGRIC, Teagasc, Moorepark, Fermoy, Co. Cork, Ireland; 2 School of Veterinary Medicine, University College Dublin, Dublin 4, Ireland; 3 Sligo RVL, Department of Agriculture, Food and the Marine, Doonally, Co. Sligo, Ireland; 4 Department of Virology, National Veterinary Research Institute, Puławy, Poland

**Keywords:** Schmallenberg virus, Cattle, Surveillance, Serology, *Culicoides* spp, Ireland

## Abstract

**Background:**

Schmallenberg virus (SBV) emerged in northern-Europe in 2011 resulting in an epidemic of ruminant abortions and congenital malformations throughout the continent. In the years following the epidemic there have been reports of SBV overwintering and continued circulation in several European countries. When the population-level of immunity declines in exposed regions, re-introduction of SBV could result in further outbreaks of Schmallenberg disease. The aims of this study were to determine the SBV seroprevalence in previously exposed Irish dairy herds in 2014 and to investigate if SBV continued to circulate in these herds in the three years (2013–2015) following the Irish Schmallenberg epidemic.

Whole-herd SBV serosurveillance was conducted in 26 herds before (spring) and following the 2014 vector-season (winter), and following the 2015 vector-season (winter). In spring 2014, 5,531 blood samples were collected from 4,070 cows and 1,461 heifers. In winter 2014, 2,483 blood samples were collected from 1,550 youngstock (8–10 months old) and a subsample (n = 933; 288 cows, 645 heifers) of the seronegative animals identified in the spring. Youngstock were resampled in winter 2015. *Culicoides* spp. were collected in 10 herds during the 2014 vector-season and analysed for SBV; a total of 138 pools (3,048 *Culicoides*) from 6 SBV vector species were tested for SBV RNA using real-time PCR.

**Results:**

In spring 2014, animal-level seroprevalence was 62.5 % (cows = 84.7 %; heifers = 0.6 %). Within-herd seroprevalence ranged widely from 8.5 %–84.1 % in the 26 herds. In winter 2014, 22 animals (0.9 %; 10 cows, 5 heifers, 7 youngstock) originating in 17 herds (range 1–4 animals/herd) tested seropositive. In winter 2015 all youngstock, including the 7 seropositive animals in winter 2014, tested seronegative suggesting their initial positive result was due to persistence of maternal antibodies. All of the *Culicoides* pools examined tested negative for SBV-RNA.

**Conclusions:**

SBV appears to have recirculated at a very low level in these herds during 2013 and 2014, while there was no evidence of SBV infection in naïve youngstock during 2015. A large population of naïve animals was identified and may be at risk of infection in future years should SBV re-emerge and recirculate as it has done in continental Europe.

## Background

Schmallenberg virus (SBV) is a novel Orthobunyavirus (family *Bunyavirudae*) which was first identified at the Friedrich Loeffler Institute in Germany in October 2011 by metagenomic analyses of blood samples collected from dairy cattle presenting non-specific clinical signs [1]. Cattle, sheep and goats are the main species affected by the virus; however SBV has also been detected in other ruminants including camelids and deer [2]. The virus is transmitted by a range of *Culicoides* spp. biting midges [3, 4]. Infection in adult cattle is typically mild; clinical signs include short-duration fever, diarrhoea and a drop in milk yield in dairy cows [1]. If susceptible ruminants become infected with SBV during the gestation-susceptible period (estimated to be between day 62–173 in cattle and day 28–56 in sheep) [2], trans-placental foetal infection can occur resulting in abortions, stillbirths and congenital malformations of the musculoskeletal and central nervous system (arthrogryposis-hydranencephaly syndrome) in neonates [4].

Following the initial emergence of SBV in Germany in 2011, the virus rapidly disseminated throughout the continent reaching an almost pan-European distribution by the end of the 2011–2012 vector-season [5] with herd seroprevalence rates nearing 100 % in many regions [2, 4]. The first Irish SBV case confirmed by RT-PCR was in a bovine foetus with pathognomonic SBV lesions in October 2012 [6]. National cattle SBV sero-surveys conducted in Ireland at the end of 2012 and in 2013 demonstrated that much of the south and south east of the country had been exposed to SBV during 2012, while the north and north-west remained predominantly uninfected [7]. There was little evidence of further spread of SBV (expansion of the area exposed to SBV) in Ireland over the course of the second vector season (2013) [7]; this is in contrast with other European countries such as Germany [8] and Belgium [9], where SBV appeared to re-emerge in cattle herds and sheep flocks in 2012. In the three years (2012, 2013 and 2014) following the European Schmallenberg epidemic there have been a number of reports of SBV overwintering and continued virus circulation in several countries [10–15]. Currently (2016), it is unknown whether SBV continues to be present and active in previously exposed regions in Ireland.

Cattle are the preferred host for the most common *Culicoides* arbovirus vector species [16, 17] and are often used as sentinel animals in surveillance programs for vector-borne diseases [18, 19]. Hence, simultaneous monitoring of *Culicoides* and their preferred bovine host species for evidence of infection is an established method to investigate arbovirus circulation in a region. The aims of the present study were (i) to determine the seroprevalence of SBV infection in 26 previously exposed Irish dairy herds in 2014 (two years after SBV first emerged in Ireland in 2012) and (ii) to investigate whether there was evidence of SBV circulation in these herds in the three years (2013–2015) following the Irish Schmallenberg epidemic, and if so to what extent.

## Methods

### Sampling design

**Fig. 1:**
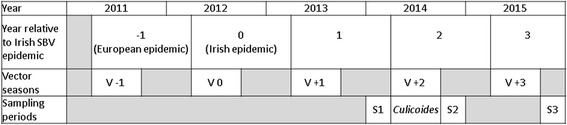
Sampling design. This SBV surveillance study was conducted in the three years following the 2012/2013 Irish Schmallenberg epidemic; Year +1, Year +2, Year +3 correspond to years one, two and three after the Irish epidemic, respectively. Surveillance years correspond with the beginning of the vector-season (V-1 to V + 3) and end with the drop in the peak of congenital malformations (end of spring calving period) associated with that particular vector-season. Blood samples were collected during three sampling periods (S1, S2, and S3); before (S1) and after (S2) the 2014 vector-season and after the 2015 vector-season. *Culicoides* spp. were collected on farms during the 2014 vector-season, (V +2)

For the purpose of this study the timespan before, during and after the Irish Schmallenberg epidemic was divided into five time periods; Year −1 to Year +4. Year −1 is the year prior to SBV emergence in Ireland (and the year of the European Schmallenberg epidemic, 2011/2012), Year 0 is the year of the Irish epidemic (2012/2013) and Year +1, Year +2 and Year +3 are one, two and three years after the Irish epidemic, respectively. Each year-long period ranges from May until April of the following year (Fig. 1). This period corresponds with the start of the vector-active season (May), [20], and the end of the peak period of congenital Schmallenberg malformations (end of peak spring calving period in April) associated with that particular vector-active season (1 May- 1 November) [20].

**Fig. 2:**
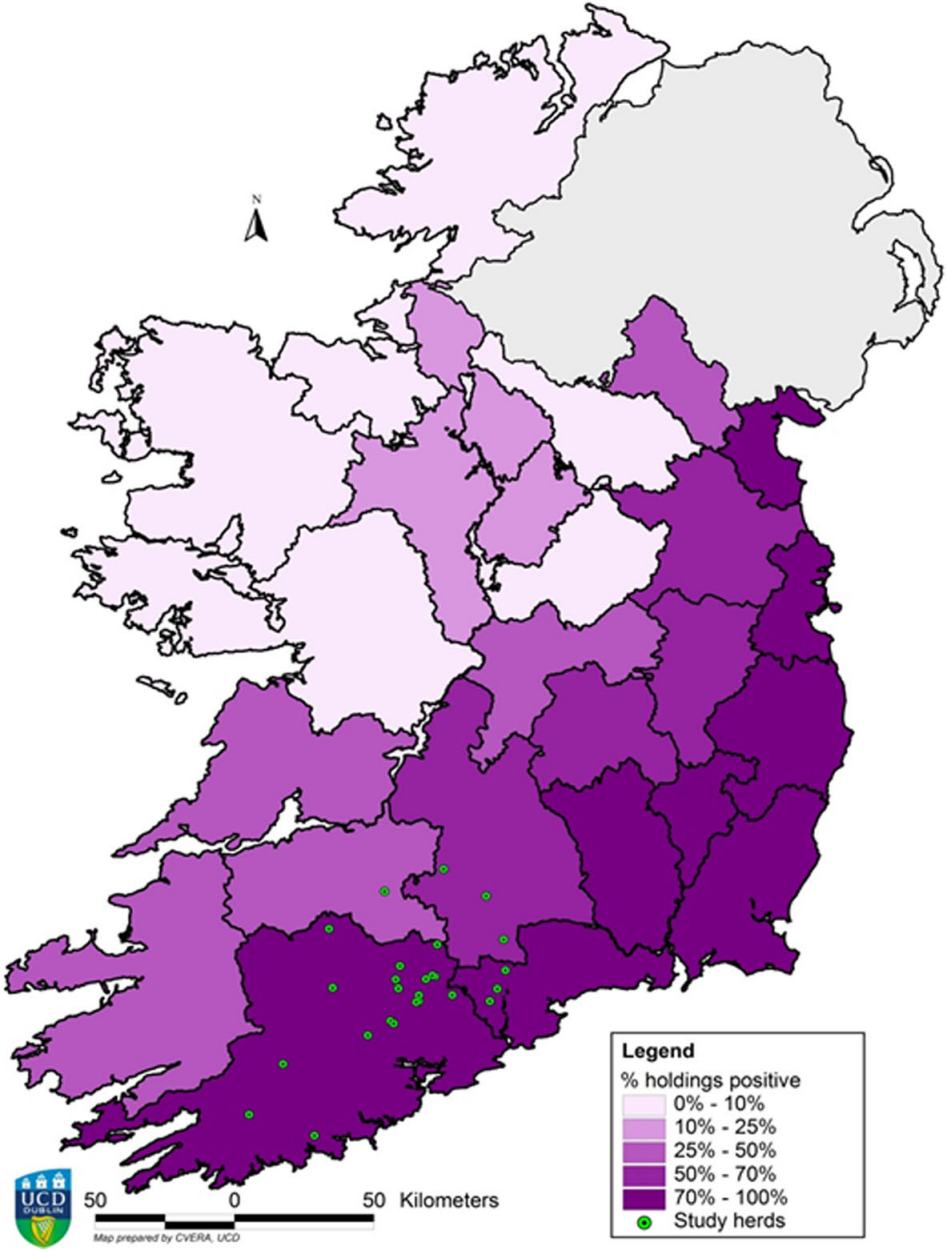
Herd locations. Spatial distribution of study surveillance herds in a region exposed to SBV during the 2012/2013 Irish Schmallenberg epidemic, including county-level percentage of SBV antibody positive herds in Ireland in December 2013 [7]. The original published map has been modified to include the present study herd locations and county-level percentage of SBV antibody-positive herds only

In order to investigate the prevalence of SBV infection and virus re-circulation, we monitored cattle herds and SBV *Culicoides* vector species for evidence of infection in the three years (2013, 2014 and 2015) following the Irish Schmallenberg epidemic. In 2014, a total of 36 Irish dairy farmers located in a region where SBV circulated extensively during the Irish Schmallenberg epidemic [7] were invited to participate in an epidemiological study on SBV surveillance. These herds were selected from a sampling frame of 111 farmers for a separate calf mortality study [21]. These herds were screened for spring-calving (January–May), more than 50 calvings/year, calf mortality rate, farmers with a history of good record keeping and farm located within a 1.30 h driving time radius of Moorepark. Twenty-six of the 36 dairy herds distributed at random in an SBV exposed region (Fig. 2) were successfully enrolled in the two year study which commenced in spring 2014.

A total of 7,081 animals (n = 9,454 blood samples) were evaluated for evidence of SBV infection during the study period (Table 1). Blood samples were collected during three sampling periods; S1 (spring 2014: 18th March – 5th April), S2 (winter 2014: 1st November–11th December) and S3 (winter 2015: 18th November–10th December). *Culicoides* were collected during the 2014 vector-active season (V +2).

**Table 1:** Number of animals blood sampled in each animal group

Animal type	Sampling period 1	Sampling period 2	Sampling period 3
	Spring 2014	Winter 2014	Winter 2015
Lactating cows	4070	288	n/s
Heifers	1461	645	n/s
Youngstock	n/s	1550	1440
Total	5531	2483	1440

(*n/s* not sampled)

During the first sampling period (S1) whole-herd SBV serology was conducted in order to determine animal-level and herd-level SBV seroprevalence. A total of 5,531 individual animal blood samples were collected from 4,070 cows (born in 2012 and earlier) and 1,461 replacement heifers (born in spring 2013) in 26 study herds (range 73–561 animals/herd) and evaluated for SBV-specific antibodies. This sample size was sufficient to estimate animal-level SBV true seroprevalence in this population of animals with 99 % confidence and 1 % precision using *a priori* herd prevalence estimate of 60 % in cattle herds in this region after the 2013 vector-season [7, 22] and ELISA test sensitivity and specificity of 97.6 and 100 %, respectively (ID Screen® internal validation report) [22, 23].

During the second sampling period (S2), a subsample of the seronegative animals identified in spring 2014 (n = 933; range 6–87 animals/herd) were resampled in each herd after the 2014 vector-season (V +2) and evaluated for evidence of SBV infection during 2014. This sample size was sufficient to demonstrate freedom of SBV circulation in these animals in 2014 with 99 % confidence using the present study spring 2014 heifer estimated true prevalence results of 0.6 % (99 % CI: 0.2–1.4 %).

Blood samples were collected from 1,550 youngstock (range 25–118 animals/herd) from 25 of the present study herds (one herd opted out of the study) in winter 2014 (S2) and monitored prospectively for one year. Youngstock were resampled in winter 2015 (S3) and evaluated for SBV exposure during 2015. For this evaluation it was assumed that the duration of persistence of maternal antibodies was between 6–8 months [24]; hence, only animals between 8–10 months of age were included in the study. This sample size was sufficient to demonstrate freedom of SBV circulation in these herds in 2015 with 99 % confidence using our winter 2014 youngstock estimated true prevalence results of 0.5 % (99 % CI: 0–0.9 %).

Serum samples were analysed for SBV-specific antibodies using a competitive ELISA (ID Screen Schmallenberg virus Competition Multi-species, IDVet, Montpellier, France) in accordance with manufacturer’s guidelines. The assay specificity and sensitivity were reported by the manufacturer at 100 % (95 % CI: 99.64–100 %) and 97.6 %, respectively (manufacturer internal validation report). Optical density (OD) values measured by the ELISA reader were transformed to sample-to-negative percentage ratios (S/N %) using the manufacturer’s set of positive and negative controls using the formula: S/N% ratio = (OD _Sample_ / OD _Negative control_) × 100. Serum samples with an S/N% value less than or equal to 40 %, greater than 40 % and less than or equal to 50 %, and greater than 50 %, were considered as positive, inconclusive and negative, respectively.

### Culicoides collection

Ultraviolet light trapping for *Culicoides* spp. was carried out on 10 of the study farms during the 2014 vector-active season (V2) as part of a separate entomological study investigating the species, abundance and ecological habitats of biting midges in Irish cattle herds, and their potential role as arbovirus vectors [25]. Each site was sampled fortnightly over a period of 16 weeks from 21^st^ July to 5^th^ November 2014; one Onderstepoort Veterinary Institute UV light trap (OVI trap) was run overnight in the vicinity of livestock. Catches were transferred immediately into 80 % ethanol. A total of 24,094 *Culicoides* were caught during the sampling period which included 6,621 pigmented (or so called *parous*; ovoposited and blood-fed at least once) *Culicoides* from six known SBV vector species *(C. obsoletus/scoticus, C. pulicaris, C. punctatus, C. dewulfi, C. chiopterus*). The midges were morphologically identified to species level and parity status using the keys of Campbell and Pelham-Clinton (1960) [26] and pooled accordingly.

Pools comprising approximately 23 pigmented midges from the six known SBV vector species were prepared for PCR analysis; each pool contained specimens from each catch collection for one specific site. Sample size calculation for the demonstration of freedom (detection of disease) in *Culicoides* using pooled testing was calculated using a design prevalence of 0.15 %; this figure was estimated from a review of published reports of SBV detection in field-caught *Culicoides* between 2011 and 2012 in Belgium, Denmark, Netherlands, Italy, Poland and France [4] and in field-caught *Culicoides* in Poland between 2013 and 2015 (Larska et al., unpublished observations) as the prevalence of SBV in Irish *Culicoides* spp. is unknown. Real time rt-PCR test sensitivity was assumed to be 100 % for the purpose of this sample size calculation as no data are available defining the rt-RT-PCR test sensitivity for the detection of SBV RNA in C*ulicoides* insect specimens.

Hence, a sample size of 138 pools (n = 3,043 pigmented *Culicoides*) was used to detect SBV infection on these farms during the sampling period with 95 % confidence. The number of pools for each species tested was determined from the *Culicoides* spp. prevalence on these farms; the most abundant species identified were *C. obsoletus/scoticus* complex (37.6 %; n = 46 pools) and *C. dewulfi* (35.9 %; n = 46 pools)*,* followed by *C. pulicaris* (9.0 %; n = 20 pools), *C. punctatus* (5.6 % n = 12 pools) and *C. chiopterus* (4.5 % n = 14 pools). The species *C. nubeculosis* were underrepresented, while the other 14 *Culicoides* spp. identified are not considered SBV vectors; these species were therefore omitted from the virological testing. *Culicoides* spp. pools were tested for SBV RNA using real-time RT-PCR, adapted from the method described previously [3]. Insects were homogenised in RLT buffer using Lysing Matrix D tubes containing ceramic beads in a ribolyzer (Thermo Electron Corp., Milford, USA) for 90 s at 6.5 m/s speed. Total RNA was purified using RNeasy Mini Kit (Qiagen®) in the Qiacube automatic station (Qiagen®). Duplex real-time RT-PCR pairs of primers designed to detect SBV S segment and the 18S midge gene fragment in *C. obsoletus/scoticus* complex*, C. chiopterus, C. pulicaris, C. punctatus* as internal control were used. For the specific detection of *C. dewulfi* RNA*,* an additional set of primers flanking a fragment of mitochondrial cytochrome oxidase 1 gene was used [27]. Samples were analysed in the in-house optimised rt-RT-PCR using AgPath-ID OneStep RT-PCR Reagents kit (Ambion, Applied Biosystem) in a StepOne Real-Time PCR system (Life Technologies) as previously described [3]. A threshold cycle (C_t_) value of ≤40 was considered the cut off value to detect SBV positive pools.

### Statistical methods

Epidemiological analyses, including apparent prevalence (AP) and estimated true prevalence (TP) rates, and sample size calculations were completed using an online epidemiological calculator (EpiTools) [22].

## Results

### Spring 2014 (S1)

Seroprevalence results are shown in Table 2. In total, 3,375 animals tested positive for SBV-specific antibodies, which gave an animal-level AP of 61.0 % (CI 99 %: 59.3–62.6 %) and TP of 62.5 % (99 % CI: 60.8–64.2 %) when correcting for imperfect test characteristics. Seropositive animals were present in all 26 herds. Within-herd TP ranged widely from 8.5 to 84.1 % (median 70.1 %) across the 26 herds. In cows, animal-level AP was 82.7 % (99 % CI: 81.1–84.2 %), TP was 84.7 % (99 % CI: 83.1–86.3 %) and ranged between 10.7 % and 100 % across the 26 herds. In heifers, animal-level AP was 0.6 % (99 % CI: 0.3–1.4 %) and TP was 0.6 % (99 % CI: 0.2–1.4 %). Seropositive heifers (n = 9) originated in 8 herds and ranged from 1–2 animals per herd (Table 3). Of all the seronegative animals identified in spring 2014, 68.5 % were spring 2013-born heifers; of which 98.8 % were seronegative. Inconclusive blood results were re-tested and confirmed using the same ELISA testing kit.

**Table 2:** Animal-level and within herd SBV seroprevalence in previously exposed Irish dairy herds in 2014 and 2015, with number of positives animals and respective TP (true prevalence) rates in each herd/animal group

	Sampling period 1					Sampling period 2						Sampling period 3	
	Spring 2014					Winter 2014						Winter 2015	
	Whole herd					Seronegative animals				Youngstock		Youngstock	
Herd ID	Herd size	No. of positives	Herd TP (%)	Cows TP (%)	Heifers TP (%)	Sample size	No. of positives	Cows positive	Heifers positive	Herd size	No. of positives	Sample size	No. of positives
1	148	101	69.9	99.5	2.3	Farmer opted out of study							
2	390	198	52	72.2	0	86	1	0	1	99	0	94	0
3	180	15	8.5	10.7	2.2	75	0	0	0	30	0	29	0
4	196	138	72.1	96.2	0	30	1	0	1	44	1	43	0
5	117	85	74.4	94.7	0	16	1	1	0	20	0	20	0
6	106	79	76.4	92	0	9	1	0	1	75	0	66	0
7	284	166	59.9	71.2	0	55	3	2	1	28	0	28	0
8	169	119	72.1	91.7	0	14	0	0	0	33	0	32	0
9	73	58	81.4	100	0	8	0	0	0	20	0	20	0
10	241	27	11.5	16.2	0	87	0	0	0	79	0	77	0
11	142	59	42.6	63	0	36	0	0	0	39	0	23	0
12	379	261	70.6	95.8	1	52	2	2	0	106	0	105	0
13	338	232	70.3	99.9	1	53	1	0	1	106	1	106	0
14	123	88	73.3	100	0	18	1	1	0	36	0	35	0
15	283	180	77.5	99.2	0	43	0	0	0	84	1	81	0
16	561	435	79.4	100	0	55	2	2	0	118	1	115	0
17	338	209	63.4	96.5	0	50	1	1	0	77	1	76	0
18	217	143	67.5	90.4	0	34	0	0	0	81	1	62	0
19	206	169	84.1	98.9	0	17	0	0	0	50	0	49	0
20	163	94	59.1	89.1	1.8	32	0	0	0	81	0	76	0
21	89	65	74.8	95	5.1	6	0	0	0	34	0	9	0
22	212	117	56.5	77.5	3.4	50	1	1	0	66	1	65	0
23	106	66	63.8	96.6	0	19	0	0	0	44	0	41	0
24	218	154	72.4	99.9	0	27	0	0	0	115	0	114	0
25	113	26	23.6	30.6	0	37	0	0	0	35	0	30	0
26	139	91	67.1	100	2.1	24	0	0	0	50	0	44	0
Total	5531	3375	62.5	84.7	0.6	933	15	10	5	1550	7	1440	0

**Table 3:** SBV ELISA-positive animals detected in surveillance Year +1, Year +2 and Year +3 in cows (C), heifers (H), youngstock (Y) in each herd, with the number of positive animals detected per herd in each surveillance year, blood sampling dates, ELISA S/N% results, Positive (P), Negative (N), S/N% value from the ELISA threshold cut-off and date of birth and age (months) of animals at first blood sampling

Surveillance year	Herd ID	Animal type	Number of positives	Blood sample 1						Blood sample 2			
				Date	ELISA S/N %	Result	S/N% from cut off	Date of birth	Age (months)	Date	ELISA S/N %	Result	S/N % from cut off
Year +1 (2013)^a^	1	H	1	29-Mar-14	6.9	P	33.1	15-Mar-13	31.2	–	–	–	–
	3	H	1	28-Mar-14	4.9	P	35.1	13-Mar-13	31.3	–	–	–	–
	12	H	1	21-Mar-14	25.0	P	15.0	07-Mar-13	31.2	–	–	–	–
	13	H	1	21-Mar-14	32.8	P	7.2	14-Feb-13	33.1	–	–	–	–
	20	H	1	14-Mar-14	37.6	P	2.4	15-Feb-13	32.4	–	–	–	–
	21	H	1	28-Mar-14	33.1	P	6.9	16-Mar-13	31.0	–	–	–	–
	22	H	2	22-Mar-14	31.4	P	8.6	26-Mar-13	29.7	–	–	–	–
	22	H	2	22-Mar-14	7.6	P	32.4	26-Mar-13	29.7	–	–	–	–
	26	H	1	26-Mar-14	6.6	P	33.4	18-Feb-13	33.2	–	–	–	–
Year +2 (2014)	4	C	2	19-Mar-14	57.8	N	7.8	–	–	25-Nov-14	37.3	P	2.7
	7	C	3	21-Mar-14	64.1	N	14.1	–	–	28-Nov-14	33.9	P	6.1
	7	C	3	24-Mar-14	76.9	N	26.9	–	–	17-Nov-14	11.5	P	28.5
	12	C	2	19-Mar-14	50.7	N	0.7	–	–	27-Nov-14	23.2	P	16.8
	12	C	2	18-Mar-14	55.0	N	5.0	–	–	27-Nov-14	9.5	P	30.5
	14	C	1	21-Mar-14	50.7	N	0.7	–	–	01-Dec-14	30.4	P	9.6
	16	C	2	28-Mar-14	68.9	N	18.9	–	–	14-Nov-14	19.6	P	20.4
	16	C	2	27-Mar-14	51.0	N	1.0	–	–	20-Nov-14	30.5	P	9.5
	17	C	1	21-Mar-14	54.2	N	4.2	–	–	28-Nov-14	34.0	P	6.0
	22	C	1	18-Mar-14	77.4	N	27.4	–	–	27-Nov-14	7.2	P	32.8
	2	H	1	27-Mar-14	69.2	N	19.2	–	–	20-Nov-14	13.3	P	26.7
	4	H	1	27-Mar-14	81.1	N	31.1	–	–	20-Nov-14	23.1	P	16.9
	6	H	1	21-Mar-14	71.6	N	21.6	–	–	28-Nov-14	39.5	P	0.5
	7	H	3	14-Mar-14	72.4	N	22.4	–	–	24-Nov-14	34.6	P	5.4
	13	H	1	19-Mar-14	78.1	N	28.1	–	–	27-Nov-14	15.0	P	25.0
Year +3 (2015)	4	Y	1	24-Nov-14	38.8	P	1.2	19-Jan-14	10.17	17-Nov-15	74.4	N	24.4
	13	Y	1	28-Nov-14	34.0	P	6.0	17- Feb-14	9.37	02-Dec-15	86.0	N	36.0
	15	Y	1	19-Nov-14	34.7	P	5.3	14-Mar-14	8.17	08-Dec-15	105.4	N	55.4
	16	Y	2	27-Nov-14	32.5	P	7.5	13-Feb-14	9.47	25-Nov-15	71.7	N	21.7
	16	Y	1	27-Nov-14	37.6	P	2.4	19-Feb-14	9.27	25-Nov-15	79.7	N	29.7
	18	Y	1	12-Dec-14	36.0	P	4.0	30-Mar-14	8.40	20-Nov-15	85.7	N	35.7
	22	Y	1	01-Dec-14	34.2	P	5.8	05-Mar-14	8.87	09-Dec-15	95.8	N	45.8

^a^Surveillance year +1 = positive heifers only; positive cows are excluded as they are likely to have been exposed to SBV during the 2012 Irish SBV epidemic

### Winter 2014 (S2)

Of the 933 seronegative animals re-sampled in winter 2014, 891 [95.5 %] animals (268 [28.7 %] cows and 623 [66.8 %] heifers) remained seronegative after the 2014 vector-season. Fifteen animals (10 cows and 5 heifers) originating in 11 herds (1–3 animals per herd) tested positive for SBV-specific antibodies resulting in animal-level AP of 1.6 % (99 % CI: 0.8–3.1 %) and TP of 1.6 % (99 % CI: 0.08–3.0 %) in this group of animals. Six out of these 15 seropositive animals had an S/N% result near the ELISA S/N% cut-off threshold (Table 3). Of the 1,550 youngstock blood sampled, a total of seven animals originating in seven herds tested positive for SBV-specific antibodies (Table 3) resulting in an animal-level AP of 0.5 % (99 % CI: 0.2–1.1 %) and TP of 0.05 % (99 % CI: 0.01–1.1 %) in youngstock. Nineteen (1.23 %) animals had inconclusive results. Positive and inconclusive blood results were re-tested and confirmed using the same ELISA testing kit.

### Winter 2015 (S3)

In winter 2015, 1,440 youngstock were resampled; 110 animals originating in 25 herds (range 1–25 animals per herd) had been culled/sold prior to the sampling date. All youngstock blood sampled, including the 7 animals with a positive ELISA test result in winter 2014, had a negative ELISA result when retested in winter 2015 (Table 3).

### SBV analysis of Culicoides spp

A total of 3,043 pigmented *Culicoides* (138 pools) were analysed for the presence of SBV RNA. Seven pools (two *C. obsoletus/scoticus,* three *C. chiopterus,* two *C. punctatus*) containing a total of 84 specimens (range 3–24 specimens per pool) had a negative internal control (IC) reading and were excluded from the analysis. Of the 131 pools successfully analysed all tested negative for SBV RNA; one *C. dewulfi* pool was weakly positive on initial first test (SBV C_t_ = 38.11, IC C_t_ = 19.15) but was negative when retested using rt-RT-PCR and was negative on gel electrophoresis. Internal control C_t_ values for the 131 pools evaluated ranged from 19.4 to 29.28.

## Discussion

The first incursion of SBV into Ireland occurred in August 2012 [28] which resulted in an epidemic of SBV-associated congenital malformations and abortions in cattle and sheep in autumn 2012 and spring 2013. In spring 2014, one year after the Schmallenberg epidemic in Ireland, animal-level seroprevalence in the present study herds was high [62.5 %; 99 % CI: 60.8–64.2 %]. Interestingly, almost all (99.7 %) of the seropositive animals detected in these herds in spring 2014 were cows; animal-level seroprevalence was significantly higher in cows [84.7 % (CI: 99 %: 83.1–86.3 %)] when compared to heifers [0.6 % (99 % CI: 0.2–1.4 %)] suggesting that cows were most likely exposed to SBV during the 2012 vector-season. Seropositive heifers identified in spring 2014 were most likely exposed to SBV during the 2013 vector-season. However, the possibility of in-utero infection of these heifers during the 2012 vector-season should also be considered; one study in Germany found that high SBV-specific antibody titres can be found in clinically healthy calves before colostrum intake [29]. The large population of seronegative heifers (born in spring 2013) identified in these herds in spring 2014 also demonstrates that there was a substantial decrease in SBV circulation in these herds during 2013 when compared to the year of the epidemic. These findings are consistent with similar surveillance studies in continental Europe in the first year following the epidemic; where seroprevalence remained high in adult cattle [2, 4] while a lower level of virus circulation was evident in youngstock in a number of regions [10, 13, 15].

Both between-herd (BH) and within-herd (WH) seroprevalence in the 26 herds ranged widely in spring 2014; BH range 8.5 to 84.1 %, and WH range 10.7 to 100 % in cows and 0 and 5.1 % in heifers. This highlights that despite high levels of virus circulation during the Schmallenberg epidemic in this region, individual herds and within-herd animal groupings in these herds, displayed widely different levels of immunity, and as a consequence, susceptibility to new infection during the 2014 vector season. The herds in the present study are all spring-calving, pasture-managed dairy herds; the habitats where cows grazed and where *Culicoides* spp. were collected in 2014 were predominantly improved grassland, which included a mix of semi-natural woodland and hedges [25]. Hence, the variation observed in BH and WH seroprevalence in these herds is unlikely to be due to differences in herd management practices (housing conditions, pasture management etc.). Rather it is likely a consequence of the distribution and extent of spread of SBV during the 2012/2013 Irish epidemic where there was a lower percentage of positive herds in the north and west of Ireland when compared to the south and the east [7]. In the present study, the herds with low WH seroprevalence were located further west in the country when compared to herds with high WH seroprevalence, which were located in the east.

In winter 2014, only 15 animals (10 cows and 5 heifers; originating in 11 herds) which were identified as seronegative in spring 2014, showed evidence of seroconversion during the 2014 vector-season. This low prevalence of SBV infection [AP = 1.6 % (99 % CI: 0.8–3.1 %) and TP = 1.6 % (99 % CI: 0.8–3.0 %)] in naïve animals in these herds after the 2014 vector-season suggests that SBV continued to circulate in these herds during 2014, albeit at a very low level. A similar pattern of continued virus circulation has been observed in several European countries in the years following the European Schmallenberg Epidemic [10–15]. It is important to note however, that these 15 seropositive animals were distributed at random across 11 herds and that the individual animal ELISA S/N% results varied widely between 0.5 and 32.8 % from the cut-off; some animals (n = 6) were near the cut-off a suggesting a ‘weak’ positive result, while the remaining animals had ‘highly’ positive serology results. The positive and inconclusive blood results in these animals were repeated and confirmed using the same ELISA test. Given the high specificity of the ELISA test used, the probability of a repeated false-positive result is close to zero, while the high sensitivity of this test demonstrates improved sensitivity for sera with low antibody titres (IDVet internal validation report).

Interestingly, when 46.0 % of the six known SBV *Culicoides* vector spp. specimens trapped in these herds during the 2014 vector-active season (which was sufficient to detect an individual SBV positive pool with 95 % confidence using an estimated prevalence of 0.15 %) were analysed for evidence of SBV infection, one *C. dewulfi* pool was weakly positive (SBV C_t_ = 38.11, IC = C_t_ 19.15) indicating that SBV may have also been present and circulating in *Culicoides* in these herds during 2014. However, this pool was negative when retested using rt-RT-PCR and was also negative on gel electrophoresis. These findings are consistent with the bovine serosurveillance work conducted on the same farms in the same year, supporting the lack of evidence of notable SBV circulation in these herds in 2014.

When all youngstock (born in spring 2014) in these herds were blood sampled in winter 2014 only 7 animals [AP of 0.5 % (99 % CI: 0.02–1.2 %) and TP of 0.5 % (99 % CI: 0.1–1.1 %)] originating in 6 herds had a seropositive result. The ELISA results in these 7 youngstock were all weakly positive; their S/N% results ranged between 1.2 and 7.5 % from the ELISA cut-off threshold suggesting that youngstock in these herds were exposed to SBV in 2014 at a low level. When the serology results collected from heifers and youngstock during the three study sampling periods were evaluated simultaneously, it revealed that in animals (n = 3011) born after the Irish Schmallenberg epidemic (born in spring 2013 or spring 2014), only 21 animals (14 heifers and 7 youngstock) originating in 17 herds (1–4 animals per herd) tested positive for SBV antibodies. This equates to an animal-level AP of 0.7 % (CI 99 %: 0.4–1.2 %) and TP of 0.7 % (CI 99 %: 0.4–1.2 %) in animals born after the Schmallenberg epidemic suggesting that SBV continued to circulate at low levels in these herds in 2013 and 2014.

When youngstock were retested in winter 2015, all animals, including the 7 seropositive animals identified in winter 2014, tested negative for SBV-specific antibodies demonstrating that SBV did not circulate in these herds in 2015. These 7 youngstock were aged between 8–10 months at first sampling; when they were retested 12 months later all 7 animals had a negative result suggesting that the initial ‘weak’ positive ELISA result was due to persistent maternally-derived SBV antibodies. Similar results were found in an SBV sero-survey of youngstock in the Netherlands where animals between the ages of 9–15 months tested positive at first sampling but subsequently had a negative result when retested a few months later [15]. There are limited data on the duration of SBV maternally-derived antibodies in calves and the half-life of SBV maternally-derived antibodies is unknown. However, a study in the Netherlands reported an average duration of 180 days (range 120–140) in 13 calves in one herd [24]. Furthermore, there are scant reports describing the course of decay of maternally-derived antibodies against other Orthobunyaviruses in ruminants [30]. These findings highlight the need for further research in this area; this information would be particularly useful when designing SBV vaccination protocols and for identifying the most appropriate age to vaccinate animals. Moreover, these results highlight that a large population of naïve animals may be at risk of infection should SBV re-circulate in Ireland in the future at it appears to have done in continental Europe [10–15]. This population of susceptible animals is likely to increase each year SBV does not circulate as older animals are replaced by naïve younger animals. A similar naïve population of animals is also likely to be increasing in the UK and in continental Europe [10, 15].

It is surprising that so few animals showed evidence of SBV infection in these herds during the study period despite the presence of a large population of susceptible animals in each herd during this time. It is possible that the high rate of herd-immunity at the beginning of the study (animal-level seroprevalence was 62.5 % in spring 2014) may have reduced the virus’s ability to circulate in this population of animals in the subsequent vector-seasons. The lack of clustering of seroconverted naïve animals within a herd is somewhat inconsistent with the transmission characteristics of SBV; SBV is typically highly efficient in spreading in herds in the presence of transmitting vector species [31]. This is due to the rapid rate of virus replication and the high probability of transmission from host to vector [32]. This is supported by high within-herd seroprevalence rates in cattle and sheep after the 2011/2012 epidemic [4]. These epidemiological characteristics of SBV result in a high basic reproductive number (R_0_), estimated to be as high as 6.2 in cattle herds [32], which reduces the probability of finding low numbers of seropositive animals once the virus has circulated in a naïve ruminant population [15]. Using this R_0_ value, it is interesting to note that the effective reproductive number (R_e_ = R_0_ × fraction of the population of susceptible animals) in animals in these herds in spring 2014 (one year after the Irish Schmallenberg epidemic) would have been greater than one (R_e_ = 2.33; R_e_ = 6.2 × 0.375) highlighting the potential for SBV to re-circulate in these herds during 2014, despite a high rate of herd immunity. In addition, variations in the genome of SBV have occurred in the time since the virus first emerged in 2011 [11, 33, 34]. It remains to be determined if these genetic variations have caused important enough mutations in the SBV genome to alter the immunogenicity or the transmissibility of the virus.

The mechanism by which SBV infection persists from season to season (overwinter) remains unclear [4]. Limited data suggest SBV may overwinter in vectors, possibly via vertical/trans-ovarial transmission [3]. This is supported by reports of SBV infections during the winter months despite very low vector activity [35]. Furthermore, it has been suggested in Germany, that atypical warm weather conditions during winter months may have facilitated low levels of vector activity and in turn enabled SBV transmission during the winter months [35]. SBV did not appear to circulate at the borders of the area affected in Ireland in 2012/2013 [7]. It has been suggested that the adverse weather conditions experienced in spring and early summer of 2013 [7, 36] were likely to have delayed resumption of midge activity at the start of the 2013 vector-season; this may have impeded SBV overwintering and continued circulation of the virus subsequently. A model which estimated the spread of SBV in Europe in 2011 demonstrated that the majority of SBV infections in the UK occurred as a result of infected midges being transported through downwind movement facilitated by prevailing winds from Europe [37]. The emergence of SBV in Ireland is likely to have occurred in a similar way. Moreover, during the present study, SBV was circulating at considerably lower levels in neighbouring European countries; in France in 2013/2014 and in the UK SBV circulated at a low intensity [12, 38]. This low level of virus circulation in neighbouring European countries coupled with unfavourable weather conditions in Ireland early in the 2013 vector-season [36] are likely to have reduced the risk of further incursions of SBV infected midges into Ireland during this time.

Arbovirus surveillance programs which solely monitor vectors (C*ulicoides* spp. and/or mosquitos) for evidence of infection are not considered the most effective programs to detect virus circulation or emergence. This may be due to lower virus detection rates (sensitivity) in insect specimens comparing to mammalian samples. The detection of SBV in midges can be ambiguous as it does not always mean the virus is present at transmissible levels; bimodal distributions of C_t_ values for SBV in *Culicoides* spp. are reported suggesting that the virus can be present in the vector at transmissible and sub-transmissible levels [3, 39]. Hence, arbovirus surveillance programs which combine serological, virological and entomological studies, such as the National Arbovirus Monitoring Program (NAMP) in Australia [18] are considered the most effective for arbovirus surveillance work.

Akabane virus (AKAV) is a Simbu serogroup Orthobunyavirus present in Africa, Asia, Oceania and the Middle East which is similar to SBV; AKAV is also transmitted by *Culicoides* spp. and can cause similar fatal congenital defects as SBV in infected foetuses (arthrogryposis-hydrancephaly syndrome) [40]. In Australia, *Culicoides* are distributed in well-defined geographical regions [41]. In these areas AKAV is endemic and virus transmission occurs frequently and usually each year resulting in most animals developing long-lasting protective antibodies prior to breeding age [40]. As a result, outbreaks of Akabane disease are rare and typically only occur when there is a disruption or alternation to the endemic cycle [40]. Changes in climatic conditions, expansion of the distribution of virus vectors [42] or the introduction of susceptible animals into an endemic region can influence disease outbreaks [43]. SBV appears to have followed a similar cyclical pattern of virus recirculation and re-emergence as AKAV, in Europe.

The present study suggests that SBV circulated at very low levels in previously exposed Irish cattle herds in 2013 and 2014. There was no evidence of virus circulation in youngstock in the same herds in 2015 and it is unlikely that SBV is circulating currently (2016). However, if SBV becomes endemic in continental Europe, which is possible considering the continued low levels of virus circulation in many European countries [8–15], SBV could re-emerge in Ireland in a similar fashion to AKAV re-emergence at the border zones of AKAV endemic regions. Periods of favourable weather conditions which could facilitate the mechanical transport of SBV-infected vectors, or the export of SBV-infected animals from endemic regions to susceptible regions, could result in SBV re-circulation and re-emergence in susceptible populations in the future. Furthermore, other arbovirus diseases, notably bluetongue virus which re-emerged in France in 2015 [44] and was recently identified as a potential risk to the UK in 2016 [45], could also emerge in Ireland in a similar way. This is likely to be driven and made possible by climate change [46].

### Implications

The results of the present study demonstrate that it would be prudent to continue monitoring immunologically naïve animals in previously exposed and unexposed regions for evidence of SBV infection. Furthermore, animal surveillance work for arboviruses such as SBV and bluetongue virus should be complemented with *Culicoides* spp. entomological surveillance work. In Australia, NAMP has proven effective in monitoring arboviruses such as Akabane, bluetongue and bovine ephemeral fever viruses [18]. This program monitors sentinel farms throughout the country (endemic areas, border regions and disease/vector free areas) on a permanent basis using serology, virus isolation and vector surveillance (*Culicoides*/mosquitos). Future arbovirus surveillance work in Ireland could be based on such a program.

## Conclusions

This SBV surveillance study indicated that SBV circulated at a very low level in previously exposed cattle herds in Ireland in 2013 and 2014. However there was no evidence of SBV circulation in youngstock in these herds in 2015. Moreover, a large population of seronegative animals was identified in these herds which may be at risk of SBV infection should SBV re-emerge or recirculate in the future.

## Abbreviations

AKAV: Akabane virus
Ap/Tp: Apparent/True prevalence
CI: Confidence interval
Ct: Cycle threshold
ELISA: Enzyme-Linked Immunosorbent Assay
IC: Internal control
OD: Optical density
OVI: Onderstepoort Veterinary Institute
RNA: Ribonucleic acid
RT-PCR: Real-time polymerase chain reaction
S/N: Sample-to-negative ratio
S1/S2/S3: Sampling period 1/ Sampling period 2/ Sampling period 3
SBV: Schmallenberg virus
Spp: Species
UV: Ultraviolet
V −1 V 0, V + 1, V + 2, V +3: 2011, 2012, 2013, 2014, 2015 vector-season
